# Draft Genome Sequence of Uncultured SAR324 Bacterium lautmerah10, Binned from a Red Sea Metagenome

**DOI:** 10.1128/genomeA.01711-15

**Published:** 2016-02-11

**Authors:** Mohamed F. Haroon, Luke R. Thompson, Ulrich Stingl

**Affiliations:** a Red Sea Research Center, King Abdullah University of Science and Technology (KAUST), Thuwal, Saudi Arabia; b Department of Pediatrics, University of California San Diego, San Diego, California, USA

## Abstract

A draft genome of SAR324 bacterium lautmerah10 was assembled from a metagenome of a surface water sample from the Red Sea, Saudi Arabia. The genome is more complete and has a higher G+C content than that of previously sequenced SAR324 representatives. Its genomic information shows a versatile metabolism that confers an advantage to SAR324, which is reflected in its distribution throughout different depths of the marine water column.

## GENOME ANNOUNCEMENT

Members of SAR324 (also known as marine group B) represent a deeply branched clade within the *Deltaproteobacteria*. They have been identified via 16S rRNA marker gene surveys at various depths of the marine water column but are most abundant in oxygen minimum zones (1). Despite their ubiquity in the marine environments, there is still no successful report of cultured isolates from this clade. Previous studies have retrieved SAR324 genomes from the sequencing of single cells and environmental DNA, but these genomes are highly fragmented and incomplete (<80%) (2–4). Genomic information indicates that SAR324 has a flexible metabolism, which includes sulfur oxidation, carbon fixation, hydrocarbon utilization, and heterotrophy (4). None of the sequenced genomes from SAR324 have originated from the Red Sea, although abundance profiles have shown that SAR324 comprises a significant proportion of its resident microbial population (L. R. Thompson, G. J. Williams, M. F. Haroon, A. Shibl, P. Larsen, J. Shorenstein, R. Knight, and U. Stingl, unpublished data). Here, we describe a near-complete SAR324 bacterium lautmerah10 genome sequence retrieved from shotgun metagenomic sequencing of the Red Sea.

Surface water samples (10 m depth) were collected from the Red Sea (18.58°N, 39.799°E) during the 2011 KAUST Red Sea expedition in the summer (L. R. Thompson et al., unpublished data). The seawater was filtered through different filter pore sizes (5.0 µm, 1.2 µm, and 0.1 µm), and genomic DNA was isolated from the 0.1-µm-pore-size filters (0.1- to 1.2-µm size fraction) using phenol-chloroform extraction, as previously described (L. R. Thompson et al., unpublished data; see also reference 5). Paired-end libraries (2 × 100 bp) were prepared using the Nextera DNA library prep kit (Illumina, Inc.) and sequenced on a HiSeq 2000 (Illumina, Inc.). Reads were quality checked and trimmed using PRINSEQ version 0.20.4 (6). Trimmed metagenome reads were assembled using IDBA-UD version 1.1.1 (7) with the pre-correction option. The SAR324 lautmerah10 genome was extracted based on tetranucleotide frequency and coverage using MetaBAT version 0.26.1 (8), using default parameters.

RAST (9) and the NCBI Prokaryotic Genome Annotation Pipeline were used for genome annotation. The final SAR324 lautmerah10 genome comprised 290 scaffolds, with a total length of 3.5 Mb, and contained 3,832 protein-coding genes, 31 tRNAs, and 1 rRNA operon. The completeness (>96%) and contamination (0%) of the draft genome were assessed using CheckM version 1.0.3 (10). The 16S rRNA gene showed 99% similarity to uncultured environmental clones retrieved from the Sargasso Sea (11). The G+C content (47%) is higher than that of previously sequenced SAR324 representatives (<42%). The annotation showed a high number of coding sequences (3,832 is the largest number of genes observed in an SAR324 genome to date) and supported a metabolically diverse metabolism consistent with other SAR324 bacteria, which includes sulfur oxidation, alkane oxidation, and the utilization of organic carbon compounds as electron donors. However, in contrast to other SAR324 genomes, the SAR324 lautmerah10 genome presented here does not contain the nitrite reductase gene and thus is likely not able to reduce nitrite.

### Nucleotide sequence accession number.

The SAR324 lautmerah10 draft genome has been deposited at GenBank under the accession no. LNZD00000000.
